# Banxia Xiexin Decoction in the treatment of Hp-associated peptic ulcer: A protocol for systematic review and meta-analysis

**DOI:** 10.1097/MD.0000000000024105

**Published:** 2021-01-15

**Authors:** Weikai Zhu, Jiyan Li, Hui Shen

**Affiliations:** a Traditional Chinese Medicine Department of the First Affiliated Hospital of Dalian Medical University; b Traditional Chinese medicine hospital of Dalian.

**Keywords:** Banxia Xiexin decoction, helicobacter pylori, meta-analysis, peptic ulcer

## Abstract

**Background:**

Studies suggested Banxia Xiexin Decoction is effective in the treatment of helicobacter pylori (HP) positive peptic ulcer. The present meta-analysis aimed at evaluating the efficacy and safety of Banxia Xiexin decoction in the treatment HP positive peptic ulcer.

**Methods:**

We will search PubMed, Web of Science, Cochrane Library, and Chinese biomedical databases from their inceptions to the November 30th, 2020. Two authors will independently carry out searching literature records, scanning titles and abstracts, full texts, collecting data, and assessing risk of bias. Review Manager 5.2 and Stata14.0 software will be used for data analysis.

**Results:**

This systematic review will determine the efficacy and safety of Banxia Xiexin decoction in the treatment HP positive peptic ulcer.

**Conclusion:**

Its findings will provide helpful evidence for the efficacy and safety of Banxia Xiexin decoction in the treatment HP positive peptic ulcer.

**Systematic review registration:**

INPLASY2020120002.

## 1 Introduction

Peptic ulcer (PU) is a common disease in gastroenterology. Helicobacter pylori (Hp) infection is an important cause of PU, which can lead to local ulceration and defect of duodenal mucosa.^[1]^ If patients can not get timely and standardized treatment, it is easy to lead to ulcer perforation, bleeding and pyloric obstruction. In serious cases, it can cause ulcer canceration and seriously threaten the health of patients. Western medicine mainly gives triple therapy based on proton pump inhibitors, but long-term use can lead to drug resistance and relapse easily after drug withdrawal.^[2]^ In recent years, it has been reported that traditional Chinese medicine can significantly reduce the ulcer surface and has a good curative effect.^[3,4]^ Banxia Xiexin decoction is a classic prescription in Treatise on Febrile Diseases (Shanghan Lun) of the Han Dynasty of China, which can be used for diseases such as functional dyspepsia, gastroesophageal reflux disease and colon cancer.^[5–6]^ Studies suggested Banxia Xiexin Decoction is effective in the treatment of Hp positive PU.^[7,8]^ However, there is no systematic review or meta-analysis providing evidence to determine whether Banxia Xiexin Decoction an ideal method to treat HP positive PU. Therefore, the present meta-analysis aimed at evaluating the efficacy and safety of Banxia Xiexin decoction in the treatment HP positive PU.

## 2 Materials and methods

This study will be conducted in accordance with the PRISMA (Preferred Reporting Items for Systematic Reviews and MetaAnalyses) guidelines and the protocol has been registered in the INPLASY (INPLASY2020120002).

### 2.1 Eligibility criteria

#### 2.1.1 Type of study

This study will only include high quality randomized controlled trials.

#### 2.1.2 Type of patients

The patients should be those who undergone Hp positive PU. We will not apply any restrictions of race, age, education background, and economic status.

#### 2.1.3 Intervention and comparison

This study will compare Banxia Xiexin Decoction combined with lansoprazole triple therapy and alone lansoprazole triple therapy for treating Hp positive PU.

#### 2.1.4 Type of outcomes

The primary outcome is Hp eradication rate. The secondary outcomes include traditional Chinese medicine syndrome score, quality of life score, gastrin level, incidence of adverse reactions and recurrence rate.

### 2.2 Search strategy

PubMed, Web of Science, Cochrane Library, and Chinese biomedical databases will be searched from their inceptions to the November 30th, 2020. We will not impose any limitations to language and publication status. The search strategy will be built with the assistance of a professional librarian. The search strategy for PubMed is shown in Table 1. Other online databases will be used in the same strategy.

**Table 1:** Search strategy sample of PubMed.

Number	Search terms
1	Peptic ulcer or gastric ulcer or duodenal ulcer
2	Banxia Xiexin decoction or banxia xiexintang decoration
3	and 1–2

### 2.3 Data extraction and quality assessment

Two authors will independently select the trials according to the inclusion criteria, and import into Endnote X9. Then remove duplicated or ineligible studies. Screen the titles, abstracts, and full texts of all literature to identify eligible studies. All essential data will be extracted using previously created data collection sheet by 2 independent authors. Discrepancies in data collection between 2 authors will be settled down through discussion with the help of another author. The following data will be extracted from each included research: the first author's surname, publication year, language of publication, sample size, sex, average age, course of disease, dosage, traditional Chinese medicine syndrome score, quality of life score, gastrin level, incidence of adverse reactions and recurrence rate. The Grading of Recommendations Assessment, Development, and Evaluation will be used to assess the quality of evidence. It contains 5 domains (bias risk, consistency, directness, precision, and publication bias). And the quality of evidence will be rated as high, moderate, low, and very low. Any disagreements between 2 investigators will be solved through discussion or consultation by a 3rd investigator.

### 2.4 Statistical analysis

The STATA version 15.1 software (Stata Corporation, College Station, TX) will be used for meta-analysis. We calculated the pooled summary odds ratio (OR) and its 95% confidence interval (CI). The Cochran *Q*-statistic and *I*^2^ test will be used to evaluate potential heterogeneity between studies.^[9]^ If the *Q*-test shows a *P *< .05 or I^2^ test exhibits >50%, indicating significant heterogeneity, and the random effect model will be employed or if heterogeneity is not significant, the fixed-effects model was used. If it is possible, we will perform meta-analysis to analyze the pooled outcome data when acceptable homogeneity has been identified. Otherwise, we will conduct subgroup analysis to investigate potential causes for substantial heterogeneity among eligible studies. Sensitivity analysis will be performed to evaluate the influence of a single study on the overall estimate. We will use Begger funnel plots and Egger linear regression test to investigate publication bias.^[10]^

### 2.5 Ethics and dissemination

We will not obtain ethic documents because this study will be conducted based on the data of published literature. We expect to publish this study on a peer-reviewed journal.

## 3 Discussion

Banxia Xiexin decoction consists of Banxia (Rhizoma Pinelliae), Huangqin (Radix Scutellariae), Ganjiang (Rhizoma Zingiberis), Renshen (Radix Ginseng), Zhigancao (Radix Glycyrrhizae Uralensis), Huanglian (Rhizoma Coptidis), Dazao (Fructus Jujubae). Modern pharmacological studies have shown that Banxia xiexin Decoction can protect gastric mucosa by inhibiting Hp and lowering the level of TNF-a in serum.^[11]^ Clinic Studies suggested Banxia Xiexin Decoction is effective in the treatment of HP positive PU. However, no study has published focusing on the systematic and comprehensive summary of the existing clinical evidence, which may restrict its application. In this study, we will perform a systematic review to summarize high-quality studies and to provide evidence on the evidence-based medical support for clinical practice.

## Author contributions

**Conceptualization:** Hui Shen.

**Data curation:** Weikai Zhu.

**Methodology:** Jiyan Li.

**Writing – original draft:** Weikai Zhu.

**Writing – review & editing:** Hui Shen and Jiyan Li.

## Abbreviations

Abbreviations: Hp = helicobacter pylori, PU = peptic ulcer.
